# Supplementary material for: Geometric phase magnetometry using a solid-state spin
Source: Nat Commun. 2018 Nov 27;9:4996. doi: 10.1038/s41467-018-07489-z (PMC6258709; doi:10.1038/s41467-018-07489-z)
Supplement: Supplementary file 1 — Supplementary information [file 41467_2018_7489_MOESM1_ESM.pdf]

Supplementary Information:
Geometric phase magnetometry using a solid-state spin

K. Arai*, J. Lee*, C. Belthangady, D. R. Glenn, H. Zhang, and R. L. Walsworth#

* *These authors contributed equally to this work.*

Correspondence and requests for materials should be addressed to this author.

Supplementary Note 1 - Geometric phase magnetometry signal

The geometric-phase magnetometry protocol is characterized by a time-varying Hamiltonian with three control parameters, Rabi frequency Ω, driving field phase ρ, and external magnetic field B:

$$H(t) = \frac{\hbar}{2}\mathbf{R}(t) \cdot \boldsymbol{\sigma}. \qquad (1)$$

where $\mathbf{R}(t) = [\,\Omega\cos\rho(t),\ \Omega\sin\rho(t),\ \gamma B]$ is the Larmor vector and $\boldsymbol{\sigma} = [\sigma_x, \sigma_y, \sigma_z]$ are the Pauli matrices. At time $t = t_i$, the NV spin is prepared in a superposition of two levels: $|\psi(t_i)\rangle = \frac{1}{\sqrt{2}}(|+\rangle + |-\rangle)$. If the evolution of the Larmor vector is adiabatic, the instantaneous eigenstates depend on the Larmor vector

$$|+_{\boldsymbol{R}}\rangle = +\cos\frac{\theta}{2}|+\rangle + e^{+i\rho}\sin\frac{\theta}{2}|-\rangle. \qquad (2)$$
$$|-_{\boldsymbol{R}}\rangle = -\sin\frac{\theta}{2}|+\rangle + e^{+i\rho}\cos\frac{\theta}{2}|-\rangle. \qquad (3)$$

where θ is the polar angle between the z-axis and the Larmor vector $\mathbf{R}(t)$. During the precession, the spin state vector rotates around the Larmor vector. Thus, the spin acquires a dynamic phase ϕ_d, given by

$$\phi_d(t_i, t_f) = \int_{t_i}^{t_f} |\mathbf{R}(t')|dt' = \gamma B(t_f - t_i). \qquad (4)$$

It is clear from this expression that the dynamic phase depends on the precession time. If this precession is cyclic, namely, $\mathbf{R}(t_f) = \mathbf{R}(t_i)$, the state will also acquire a geometric phase ϕ_g. To obtain the expression for the geometric phase, we first calculate the Berry connection: $\mathbf{A}^{\pm} = i\langle \pm_{\boldsymbol{R}}|\nabla_{\boldsymbol{\lambda}}|\pm_{\boldsymbol{R}}\rangle = -\frac{1\mp\cos\theta}{2r\sin\theta}\hat{\rho}$, where $\boldsymbol{\lambda} = (\theta, \rho)$ describes the polar and azimuthal angles for the Larmor vector, and $\nabla_{\lambda} = r^{-1}\partial_{\theta}\hat{\theta} + r^{-1}\sin^{-1}\theta\,\partial_{\rho}\hat{\rho}$ is the gradient. Only the ρ component of the Berry connection is nonzero. Then, the geometric phase between two states is given by

$$\phi_g^{\pm}(t_i, t_f) = \oint_C \boldsymbol{A}^{\pm} \cdot d\boldsymbol{\lambda} = -\pi N(1 \mp \cos\theta) = \mp\frac{N}{2}\Theta \quad \mathrm{mod}\ 2\pi. \qquad (5)$$

The integration is performed along the closed path C with $\rho \in [0, 2\pi N]$, and $\Theta = 2\pi(1 - \cos\theta)$ representing the solid angle subtended by the path C.

In the Berry sequence used for geometric phase magnetometry (Fig, 1b), we insert two Berry pulses between the spin echo pulses. Let us take the entire sequence length to be T. The microwave π rotation pulse about the x-axis, $e^{-i\sigma_x\pi/2} = -i\sigma_x$, flips the $|\pm\rangle$ states, giving a minus sign in front of the phase acquired during the second half of the interaction time.

$$|\psi(T)\rangle = -\frac{i}{\sqrt{2}}\, e^{-\frac{i}{2}\left[\phi_d\left(0,\frac{T}{2}\right)-\phi_d\left(\frac{T}{2},T\right)+\phi_g\left(0,\frac{T}{2}\right)-\phi_g\left(\frac{T}{2},T\right)\right]}|+\rangle \\ -\frac{i}{\sqrt{2}}\, e^{+\frac{i}{2}\left[\phi_d\left(0,\frac{T}{2}\right)-\phi_d\left(\frac{T}{2},T\right)+\phi_g\left(0,\frac{T}{2}\right)-\phi_g\left(\frac{T}{2},T\right)\right]}|-\rangle. \qquad (6)$$

The dynamic phase cancels because $\phi_d(0, T/2) = \phi_d(T/2, T) = \gamma BT/2$. However, the geometric phase can add constructively and be doubled in magnitude by alternating the direction of Larmor vector precession between each pulse:$\phi_g(0, T/2) = -\phi_g(T/2, T) = N\Theta$. At the end of the interaction time the spin state is$|\psi(T)\rangle = -\frac{i}{\sqrt{2}}\left[e^{-iN\Theta}|+\rangle + e^{+iN\Theta}|-\rangle\right]$. The final $\pi/2$-pulse maps the phase into a population difference: $P = \cos(2N\Theta)$. In Supplementary Fig. 4, the geometric phase signal is measured in a 3D parameter space of Rabi frequency, microwave frequency detuning, and winding number.

Supplementary Note 2 - 2π phase ambiguity

The dynamic phase magnetometry signal and its derivative are expressed as

$$P_d(B) = \cos[\gamma BT], \qquad \frac{\mathrm{d}P_d(B)}{\mathrm{d}B} = \gamma T \sin[\gamma BT]\,. \qquad (7)$$

For any measured signal P_d, there are infinite degenerate magnetic field values, which are related by $B_m = B + 2\pi m(\gamma T)^{-1}$, where $m = 0, \pm1, \pm2, \ldots,$ is an integer. This degeneracy cannot be resolved by measuring the slope or adding a magnetic field offset, leading to a fundamental limit in magnetic field range. In Supplementary Fig. 5a, the dynamic phase signal is plotted in 3D as a function of magnetic field B, signal P_d, and derivative $\mathrm{d}P_d/\mathrm{d}B$. When the dynamic phase magnetometry curve is projected onto the (P_d, $\mathrm{d}P_d/\mathrm{d}B$) plane, all data points lie on a closed 1D curve, given by $P_d^2 + (\gamma T)^{-2}(\mathrm{d}P_d/\mathrm{d}B)^{-2} = 1$. Even if a magnetic field offset is added, one encounters another set of infinite degeneracies. For this reason, the degeneracy of dynamic phase magnetometry signal can be resolved only if the interaction time T is changed (for example, via a quantum phase estimation algorithm). On the (P_d, $\mathrm{d}P_d/\mathrm{d}B$) plane, this approach is understood as changing the area of the closed curve. However, changing T imposes an inevitable trade-off of magnetic field range with sensitivity.

The geometric phase signal and its derivative are

$$P_g(B) = \cos\left[4\pi N\left(1 - \frac{\gamma B}{\sqrt{(\gamma B)^2 + \Omega^2}}\right)\right]. \quad (8)$$

$$\frac{\mathrm{d}P_g(B)}{\mathrm{d}B} = \frac{4\pi N\gamma\Omega^2}{((\gamma B)^2 + \Omega^2)^{\frac{3}{2}}}\sin\left[4\pi N\left(1 - \frac{\gamma B}{\sqrt{(\gamma B)^2 + \Omega^2}}\right)\right]. \quad (9)$$

For any given value of $P_g \neq \pm 1$, there are only finite degeneracies of magnetic field values. They are related by $\frac{\gamma B_m}{\sqrt{(\gamma B_m)^2+\Omega^2}} = \frac{\gamma B}{\sqrt{(\gamma B)^2+\Omega^2}} + \frac{m}{2N}$, where m is an integer, which satisfies $|m| < 2N$. This degeneracy can be resolved by measuring $\mathrm{d}P_g/\mathrm{d}B$ because the slope decreases monotonically with increasing B across a fixed value of P_g unless $P_g = \pm 1$. This concept can also be presented clearly by plotting the geometric phase signal in 3D (Supplementary Fig. 5b). In contrast to dynamic phase, geometric phase magnetometry measurements, projected onto the (P_g, $\mathrm{d}P_g/\mathrm{d}B$) plane, do not lie on a closed 1D curve. All data points except for ($P_g = \pm 1$, $\mathrm{d}P_g/\mathrm{d}B = 0$) are spread across a 2D map and the degeneracy is resolved. Note that even if $P_g = \pm 1$ is measured, one can always add a microwave frequency detuning to look for $P_g \neq \pm 1$. In summary, the geometric phase magnetometry protocol is as follows: (Step 1) Measure the signal P_g and slope $\mathrm{d}P_g/\mathrm{d}B$. (Step 2) Identify the corresponding magnetic field value B. If the measured signal and slope leaves an ambiguity, for example ($P_g = \pm 1$, $\mathrm{d}P_g/\mathrm{d}B = 0$), add a microwave frequency offset and repeat the measurement. (Step 3) The steepest slope for high-sensitivity magnetometry is accessible by tuning a microwave frequency.

Accounting for the three NV hyperfine transitions, the dynamic phase magnetometry signal becomes:

$$P_d(B) = \frac{1}{3}\sum_{m_I=-1,0,+1}\cos[(\gamma B + m_I\omega_{HF})T] = \cos(\gamma BT)\,[1 + 2\cos(\omega_{HF}T)]. \quad (10)$$

where ω_{HF} = 2.16 MHz. The hyperfine transitions introduce an envelope modulation to P_d, which changes the area of the closed curve in (P, $\mathrm{d}P/\mathrm{d}B$), but all data points still lie on the same curve. Thus, it is still not possible to resolve the degeneracy either by measuring the slope or moving to other magnetic field values. The geometric phase magnetometry signal with three hyperfine transitions is

$$P_g(B) = \frac{1}{3}\sum_{m_I=-1,0,+1}\cos\left[4\pi N\left(1 - \frac{\gamma B + m_I\omega_{HF}}{\sqrt{(\gamma B + m_I\omega_{HF})^2 + \Omega^2}}\right)\right]. \quad (11)$$

The hyperfine transitions introduce a complicated modulation. In particular, degeneracy points can appear at $P \neq \pm 1$. However, since the data points are spread across the two-dimensional (P,

dP/dB) space, the degeneracy can always be resolved by moving to a different magnetic field value. Although it is difficult to derive an analytical expression to show 2π phase unwrapping in general, exploring the following three cases are sufficient to cover the parameter space:

- For $\gamma|B| \gg \omega_{HF}$, $P_g(B) \sim \cos\left[4\pi N\left(1-\gamma B/\sqrt{(\gamma B)^2+\Omega^2}\right)\right] + O(\omega_{HF}^2)$.
- For $\gamma|B| \sim \omega_{HF} \ll \Omega$, $P_g(B) \sim \cos[4\pi N(1-\gamma B/\Omega)](1+2\cos[4\pi N(1-\omega_{HF}/\Omega)])$.
- For $\gamma|B| \sim \omega_{HF} \geq \Omega$, approximation is difficult. However, this parameter range is of importance for neither large-field-range nor high-sensitivity magnetometry.

Supplementary Note 3 - Sensitivity and field range

To calculate the sensitivity and field range, the average change of fluorescence per measurement is recast as $\Delta FL = \alpha\beta$, where $\alpha \sim 10\%$ is the NV spin-state-dependent fluorescence contrast, and $\beta \sim 0.015$ is the average number of photons collected per measurement. The sensitivity is given by $\eta \approx (\mathrm{SNR})^{-1}|dP/dB|_{\max}^{-1}\sqrt{t_m}$, where SNR$= \Delta FL/\sqrt{\beta} = \alpha\sqrt{\beta}$ represents the signal-to-noise ratio of a single measurement, $|\mathrm{d}P/\mathrm{d}B|_{\max}$ is the maximum slope of the magnetometry curve, and $t_m \approx T$ is the measurement time. For dynamic phase magnetometry, the maximum slope is $|\mathrm{d}P_d/\mathrm{d}B|_{\max} = \gamma T$, and then the sensitivity is

$$\eta \approx \frac{1}{\gamma\alpha\sqrt{\beta}}\frac{1}{\sqrt{T}}. \qquad (12)$$

The maximum field-range is defined as the half cycle of one magnetometry oscillation:

$$\mathrm{B}_{\max} \approx \frac{\pi}{\gamma T}. \qquad (13)$$

For the geometric phase magnetometry, the maximum slope of the curve is given by $\left|\mathrm{d}P_g/\mathrm{d}B\right|_{\max} = 4\pi\gamma N\Omega^{-1}$, and then the sensitivity is

$$\eta \approx \frac{1}{\gamma\alpha\sqrt{\beta}}\frac{\Omega\sqrt{T}}{4\pi N} = \frac{1}{\gamma\alpha\sqrt{\beta}}\frac{1}{2A\sqrt{T}}. \qquad (14)$$

where $A = 2\pi N/\Omega T$ is the adiabaticity parameter evaluated at $B \approx 0$. The maximum field-range is defined at the last minimum of the chirped curve:
$\phi_g(\mathrm{B}_{\max}) = 4\pi N\left(1-\gamma \mathrm{B}_{\max}/\sqrt{(\gamma \mathrm{B}_{\max})^2+\Omega^2}\right) = \pi$. By defining a small parameter, $\epsilon \equiv \phi_g/4\pi N \ll 1$, the above equation can be solved in terms of $\mathrm{B}_{\max}$ to first order:

$$B_{\max} \approx \sqrt{2N}\Omega. \qquad (15)$$

This scaling of sensitivity and field range with control parameters for geometric-phase magnetometry is confirmed in experiments (Supplementary Fig. 6&7).

Supplementary Note 4 - Berry phase coherence theory

If the NV spin qubit interacts with an environment with random noise, the Larmor vector will experience a perturbation:

$$\mathbf{R}(t) = \mathbf{R}_0(t) + \delta\mathbf{R}(t). \qquad (16)$$

The second term, assumed to be smaller than the first term, is a classical random variable representing fluctuation of the energy splitting due to coupling to environmental noise. When the measurement is repeated, the qubit acquires a different phase each time due to the random noise. Then the system is described by a mixed state using a density matrix, which is obtained by weighting the appropriate probability p for each environmental condition, $\rho(t_f) = pU(t_i, t_f)\rho(t_i)U^{\dagger}(t_i, t_f)$, where $\rho(t_i) = |\psi(t_i)\rangle\langle\psi(t_i)|$ is the initial density matrix at time $t = t_i$ constructed from a pure initial state. In particular, the diagonal elements give the probability of occupying each state, and the off-diagonal elements represent the coherence between these states. Thus, the time-averaged coherence, which can be compared to experiments, is defined as the off-diagonal component of the density matrix averaged over many realizations: $W(t_i, t_f) = |\langle\rho_{\pm}(t_f)\rangle|/|\langle\rho_{\pm}(t_i)\rangle|$.

For magnetic field sensing, the longitudinal magnetic fluctuations are of main interest as a decoherence source, so that $\delta\mathbf{R} = (0, 0, \delta R)$ is considered in the following calculations. First, the dynamic phase fluctuation is given by

$$\delta\phi_d(t_i, t_f) = \int_{t_i}^{t_f} \frac{R_z}{R}\delta R dt'. \qquad (17)$$

Since we know that the dynamic phase is canceled by an echo operation, we consider only the fluctuation term. Next, to calculate the geometric phase fluctuation, we modify the Berry connection as $A_\rho \rightarrow A'_\rho = A_\rho + \partial_\theta A_\rho \delta\theta = \frac{1}{2}\cos\theta + \frac{1}{2}\delta(\cos\theta)$. The second term describes the fluctuation of the polar angle due to the fluctuating field δR. The cyclic path is also perturbed due to the fluctuation: $dC_\phi \rightarrow dC'_\phi = Nt^{-1}dt' + \delta Nt^{-1}dt'$. The first term corresponds to the

speed of rotation of the Larmor vector, and the second term gives the first order correction due to the fluctuation. The geometric phase fluctuation is then given by

$$\delta\phi_g(t_i,t_f) = \frac{N\pi}{t_f - t_i}\int_{t_i}^{t_f}\delta(\cos\theta)dt' = \frac{N\pi}{t_f - t_i}\int_{t_i}^{t_f}\left[\frac{R^2 - R_z^2}{R^3}\right]\delta R dt' \approx A\int_{t_i}^{t_f}\delta R dt'. \quad (18)$$

Here the definition of adiabaticity $A = \dot{\rho}\sin\theta/2R$ is used. The final state is

$$|\psi(T)\rangle = -\frac{i}{\sqrt{2}}e^{+\frac{i}{2}\left(\delta\phi_d\left(0,\frac{T}{2}\right)-\delta\phi_d\left(\frac{T}{2},T\right)\right)+\frac{i}{2}\left(\delta\phi_g\left(0,\frac{T}{2}\right)-\delta\phi_g\left(\frac{T}{2},T\right)\right)}|+\rangle$$
$$-\frac{i}{\sqrt{2}}e^{-\frac{i}{2}\left(\delta\phi_d\left(0,\frac{T}{2}\right)-\delta\phi_d\left(\frac{T}{2},T\right)\right)-\frac{i}{2}\left(\delta\phi_g\left(0,\frac{T}{2}\right)-\delta\phi_g\left(\frac{T}{2},T\right)\right)}|-\rangle. \quad (19)$$

It is important to remember that the direction of revolution is switched between the first and second Berry pulses. Finally, the coherence becomes

$$W(0,T) = \langle\exp\left[-i\frac{R_z}{R}\int_0^T \delta R f_1(T;t)\,dt - iA\int_0^T \delta R\, f_0(T;\, t)dt\right]\rangle. \quad (20)$$

where $f_n(T;t) = \sum_{k=0}^{n}(-1)^k\Theta(t_{k+1} - t\,)\Theta(t - t_k)$ is a function that characterizes the pulse sequence, Θ is the Heaviside step function, and $t_0 = 0, t_{n+1} = T$.

If the noise is assumed to follow a Gaussian distribution with a zero mean $\langle\delta R(t)\rangle = 0$, then the coherence function can be reduced to the two-point correlation function $S(t_i,t_f) = \langle\delta R(t_i)\delta R(t_f)\rangle$ using Wick's theorem: $W(0,T) \sim \langle e^{-i\delta R}\rangle \sim \exp[-\langle\delta R\delta R\rangle/2]$. Hence, the coherence can be analyzed in the frequency domain by use of the spectral density of the noise: $S(\omega) = \int_{-\infty}^{+\infty} dt e^{i\omega t}S(t)$. We also define the decoherence function as $\chi(T) = -\log W(T)$. The cross term between the dynamic and geometric part becomes zero because the sequence functions f_1 and f_0 have opposite parity. Thus, the decoherence function reduces to two terms

$$\chi(T) = \chi_d(T) + \chi_g(T). \quad (21)$$

$$\chi_d(T) = \frac{1}{2}\left(\frac{R_z}{R}\right)^2\int_0^T dt_a\int_0^T dt_b\,\langle\delta R(t_a)\delta R(t_b)\rangle f_1(T;t_a)f_1(T;t_b)$$
$$= \left(\frac{R_z}{R}\right)^2\int_0^\infty \frac{d\omega}{\pi}S(\omega)\frac{F_1(\omega T)}{\omega^2}. \quad (22)$$
$$\chi_g(T) = \frac{1}{2}A^2\int_0^T dt_a\int_0^T dt_b\,\langle\delta R(t_a)\delta R(t_b)\rangle f_0(T;t_a)f_0(T;t_b)$$

$$= A^2 \int_0^\infty \frac{d\omega}{\pi} S(\omega) \frac{F_0(\omega T)}{\omega^2}. \qquad (23)$$

Here $F_0(\omega T) = \frac{\omega^2}{2} |\mathrm{FT}(f_0)|^2 = 2\sin^2\frac{\omega T}{2}$ and $F_1(\omega T) = \frac{\omega^2}{2} |\mathrm{FT}(f_1)|^2 = 8\sin^4\frac{\omega T}{4}$ are the filter functions for geometric and dynamic phase evolution in the Berry sequence, respectively.

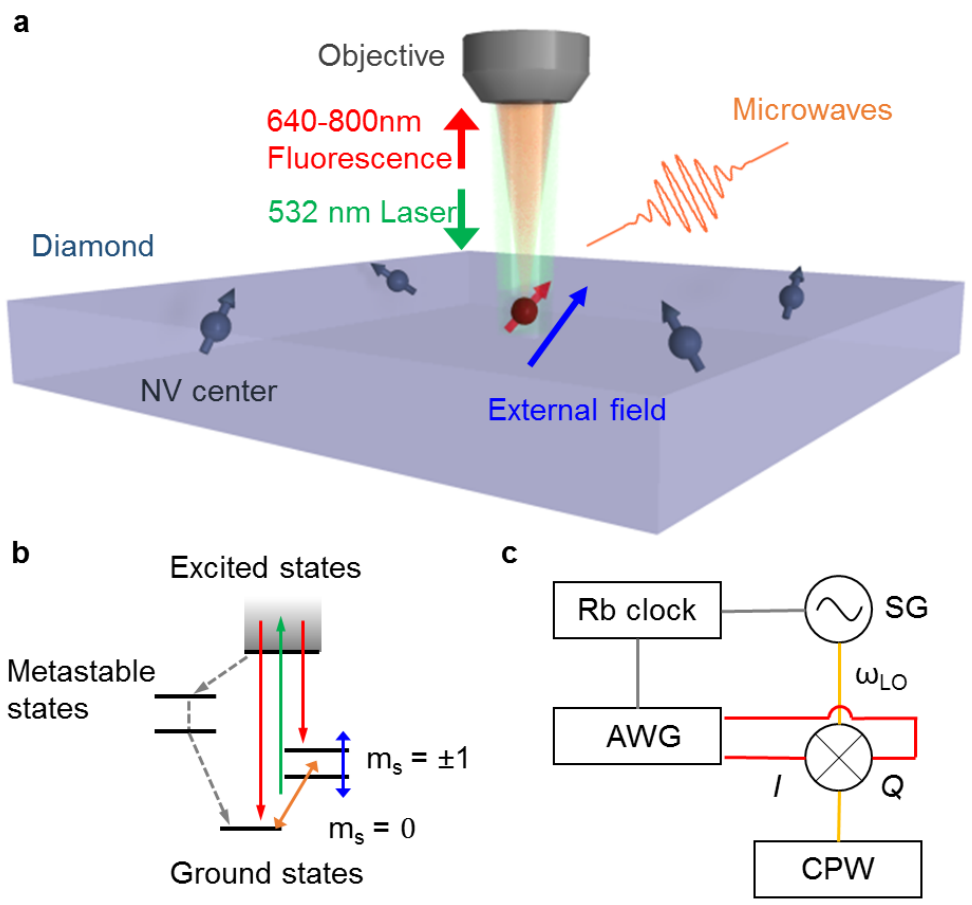

Supplementary Figure 1 | Geometric-phase magnetometry setup. a, b Schematic of geometric-phase magnetometry setup and NV optical and spin transitions. The diamond chip hosting NV centers is integrated into a confocal scanning microscope. Microwave fields (wavy orange line) produced by a gold coplanar waveguide coherently drive the $m_s = 0$ and ±1 ground states, split by $D/2\pi = 2.87$ GHz. An external magnetic field (blue arrow) created by an electromagnetic introduces Zeeman splitting between the $m_s = \pm1$ states. Excitation with 532 nm green laser causes NV spin-state dependent fluorescence in the 640-800 nm band. A nonradiative decay channel through metastable singlet states allows spin polarization as well as readout. **c,** Schematic of the microwave delivery system. A signal generator (SG, Agilent E4428C) provides the carrier microwave signal with frequency $\omega_{LO}/2\pi \sim 3$ GHz (yellow lines). The microwave pulses for geometric-phase magnetometry are generated by an arbitrary waveform generator (AWG, Tektronix AWG 5014C) and sent to the I/Q channels (red lines) of the IQ mixer (Marki IQ 1545 LMP). The output signal from the IQ mixer is delivered to a gold coplanar waveguide (CPW). To reduce phase jitter noise, an in-laboratory Rubidium clock (Stanford Research Systems FS725) phase-locks the signal generator and AWG at 10 MHz.

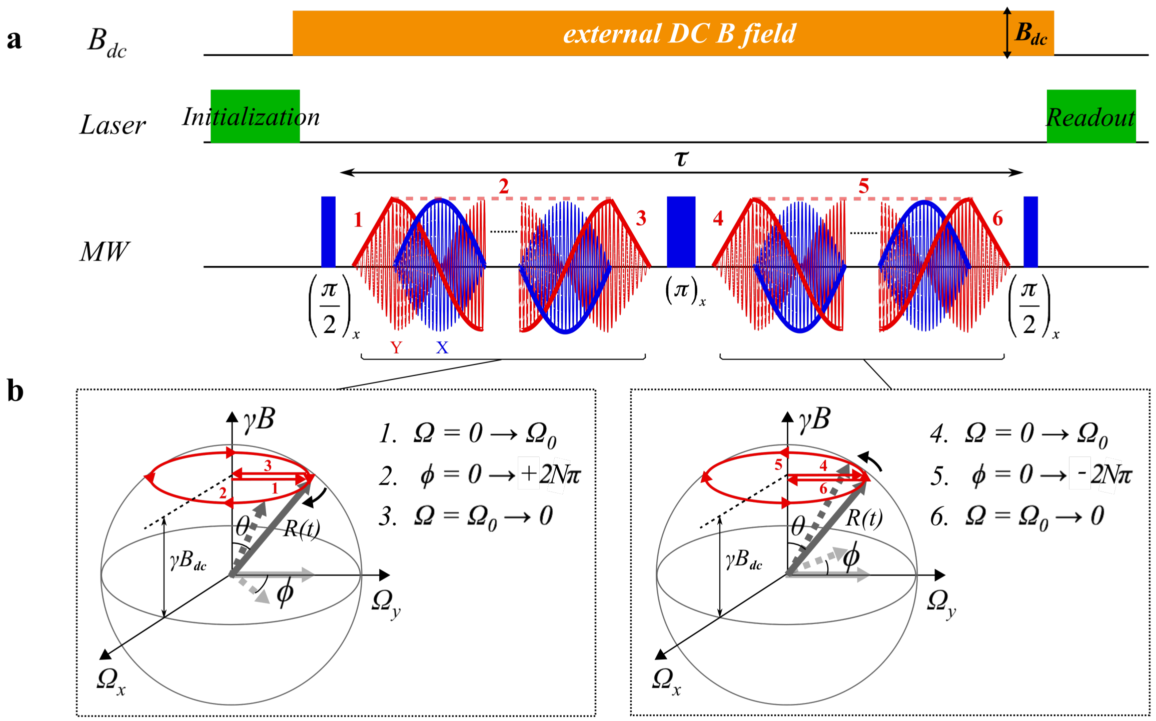

Supplementary Figure 2 | Schematic of geometric-phase magnetometry. a, Pulse sequence for geometric-phase magnetometry with an external dc magnetic field (B_{dc}) that is varied to acquire a magnetometry curve. A pulsed green laser initializes and reads out the NV spin state. A Berry sequence is applied on top of a Hahn-echo pulse sequence to cancel out the dynamic phase component of total phase accumulation. Resonant $(\pi/2)_x$ and $(\pi)_x$ pulses define a rotating frame, and B_{dc} causes a detuning of the Larmor vector **R**(t) during the Berry sequence. Phase rotation of the Larmor vector **R**(t) is controlled by two quadrature bias microwave fields (X : blue, Y : red). One sinusoidal modulation period corresponds to a full rotation (ϕ=2π) with the winding number N=1. N can be controlled by varying the sinusoidal modulation number of two quadrature bias microwaves. **b.** Steps of Berry sequence with parameter sphere picture. For the first half of the Berry sequence, with detuning of γB_{dc}, the Larmor vector **R**(t) (1) gradually gains a Rabi amplitude Ω_0, (2) rotates clockwise for ϕ=2$N\pi$, and (3) comes back to Rabi amplitude of 0. For the second half of the Berry sequence, with detuning of γB_{dc}, the Larmor vector **R**(t) (4) gradually gains a Rabi amplitude Ω_0, (5) rotates now counter clockwise for ϕ=2$N\pi$, and (6) comes back to Rabi amplitude of 0.

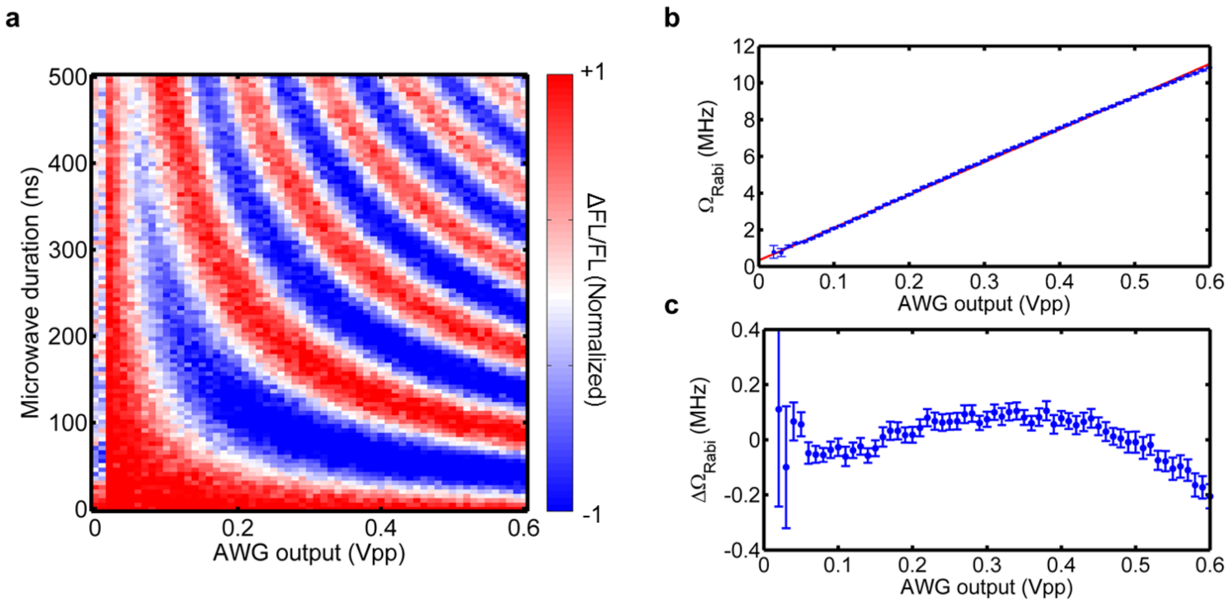

Supplementary Figure 3 | Calibration of NV Rabi frequency as a function of AWG output voltage. **a,** Normalized Rabi signal, measured as a function of the AWG output voltage and microwave pulse duration. **b,** Reduced Rabi frequency values $\Omega_{Rabi} = \Omega/2\pi$ in units of MHz (blue dots) obtained by fitting data in **a** to a sinusoidal function at each AWG voltage. Red line is a linear fit of Ω_{Rabi} to voltage. **c,** Residuals of linear fit shown in **b**. The nonlinearity of Ω_{Rabi} is less than 0.2 MHz, which can be attributed to power compression by the I/Q mixer (1 dB compression point is measured to be 1.0 Vpp). Error bars in **b**,**c** are calculated from standard error of a sinusoidal fit to the Rabi curve for each AWG output voltage.

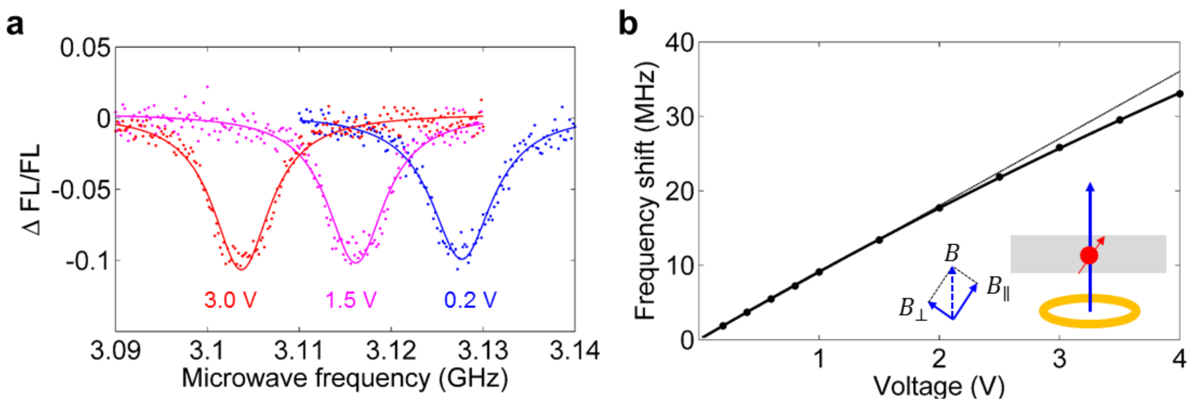

Supplementary Figure 4 | Magnetic field calibration via NV electron spin resonance. a, Continuous electron spin resonance (ESR) measurement of NV spin transitions in presence of an external magnetic field. First, to split the $m_s = \pm 1$ states, a static field of $B_0 = 93$ G is applied along the [111] NV axis using a permanent magnet, which sets the $m_s = 0, +1$ resonance peak at 3.13 GHz. Next, an additional external field B is applied using a 40-turn electromagnetic coil placed $h = 0.5$ mm above the NV center. The coil is also connected to a 150 Ω resistor in series. A high-stability voltage controller provides a high-precision electric current through the coil. An output voltage setting of 3.0 V gives $I = 0.02$ A, corresponding to a magnetic field of $B = \mu_0 NI/4\pi h \sim 16$ G in the direction perpendicular to the [100] diamond surface. NV ESR lines are measured for $V = 0.2$, 1.5, and 3.0 V (color dots). The ESR center frequency is extracted by Gaussian fitting to the date (solid lines). **b,** Absolute value of the ESR frequency shift as a function of applied voltage to the electromagnet (dots). The additional external field B has longitudinal and transverse components with respect to the NV axis: $B = B_{\parallel} + B_{\perp}$. The inset diagram indicates a side-view of the magnetometry setup, showing the diamond (gray box), NV axis (red arrow), copper electromagnet (orange circle), and direction of B (blue arrow). The NV spin state frequencies as a function of B are obtained by solving the eigenvalue problem, $\det(H - \lambda I) = 0$, where $H/\hbar = (D + \gamma B_0 + \gamma B_{\parallel})\sigma_z/2 + \gamma B_{\perp}\sigma_x/2$ is the two-level system Hamiltonian. Thus, for $\gamma B \ll D$, the measured frequency shift exhibits a quadratic term as a perturbation: $B_{\parallel} + 2\gamma B_{\perp}^2/(D + B_0)$ (thick solid curve). For very small B (few gauss), a linear fit gives a calibration of B to the applied electromagnet voltage of $\Delta B/\mathrm{V} = 0.50 \pm 0.01$ G V^{-1} (thin line).

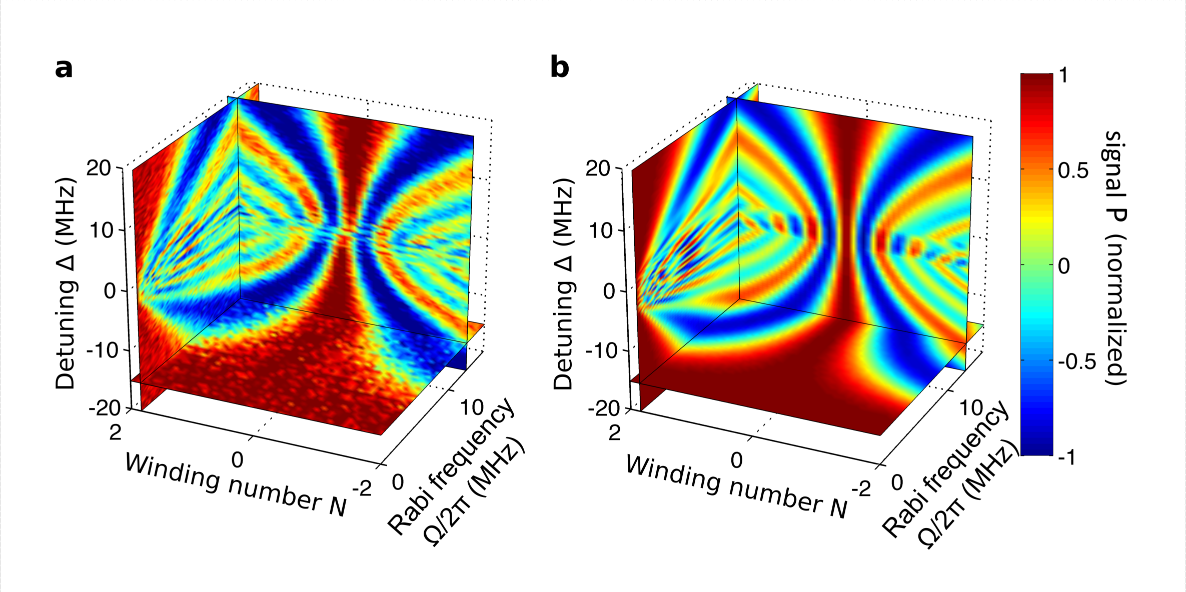

Supplementary Figure 5 | Observation of geometric phase dependence on control parameters using a single NV spin in diamond. **a**, Measurement of the cosine of the geometric phase as a function of Rabi frequency Ω, microwave frequency detuning Δ, and winding number N with a fixed interaction time of $T = 10$ μs. The amplitude of each hyperfine oscillation is extracted by fitting the data along Δ at $N = 2$, $\Omega/2\pi = 12$ MHz to an analytical expression for the geometric-phase signal including the three NV hyperfine transitions. The measured signal is normalized to the mean of these three amplitudes. **b**, Analytical model of the cosine of the geometric phase including the three hyperfine transitions, with the relative amplitudes determined in **a**. The measurement and analytical model agree well.

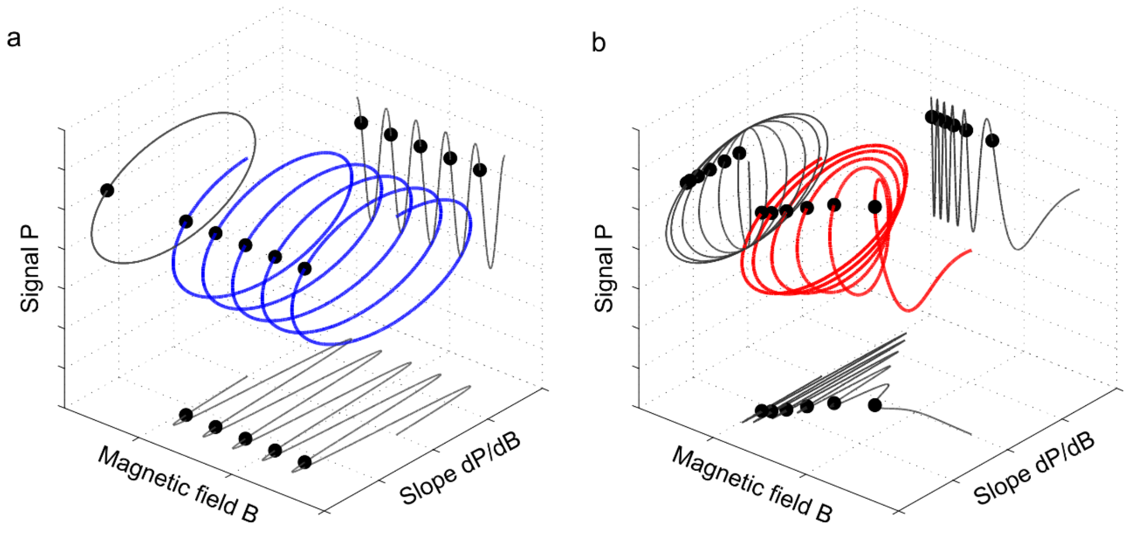

Supplementary Figure 6 | Graphical representation of 2π phase ambiguity. Magnetometry signal P and slope dP/dB are plotted against magnetic field B. Grey curves are projections. **a,** Dynamic-phase magnetometry. All data points lie on a single circle on the (P, dP/dB) plane. Thus there are infinite possibilities of B (black dots) projected onto the same point on the (P, dP/dB) plane. Adding a magnetic field offset does not solve this 2π phase ambiguity. **b**, Geometric phase magnetometry. Since P(B) is chirped, data points are distinct on the (P, dP/dB) plane, except for $P = \pm 1$. There are finite values of B (black dots) that give the same signal; but one can resolve this degeneracy by also evaluating dP/dB. For $P = \pm 1$, once can still resolve the degeneracy by adding a known magnetic field offset.

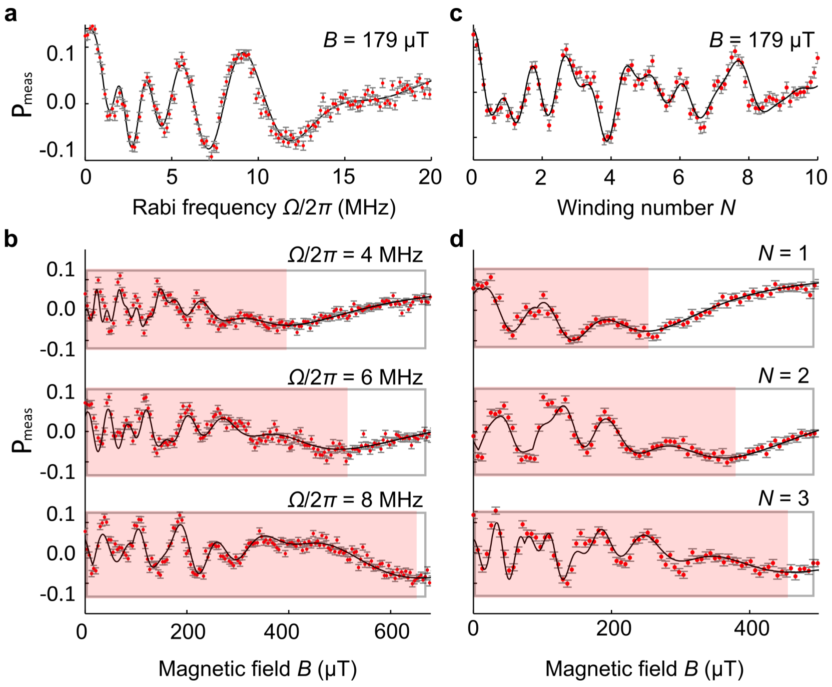

Supplementary Figure 7 | Dependence of geometric-phase magnetometry signal on control parameters. **a-d**, Geometric-phase magnetometry data (dots) for various values of Rabi frequency Ω and winding number N, as well as applied magnetic field B. Vertical axes give the measured optical signal $P_{meas} = (\Delta FL/FL)*k$, where $\Delta FL/FL$ is the fractional change of NV-spin-state-dependent fluorescence and k is a constant that depends on NV readout contrast. Error bars are one standard deviation photon shot-noise. Black lines are fits of the data to an analytical expression of the geometric-phase signal including the three NV hyperfine transitions. Red shaded regions indicate the maximum magnetic field range defined by the last oscillation minimum. Fixed parameters used in these measurements are: (**a**) B = 179 μT, N = 3, T = 10 μs, (**b**) $\Omega/2\pi$ = 4, 6, 8 MHz, N = 3, T = 10 μs, (**c**) B = 179 μT, $\Omega/2\pi$ = 5 MHz, T = 10 μs, and (**d**) $\Omega/2\pi$ = 5 MHz, N = 1, 2, 3, T = 10 μs.

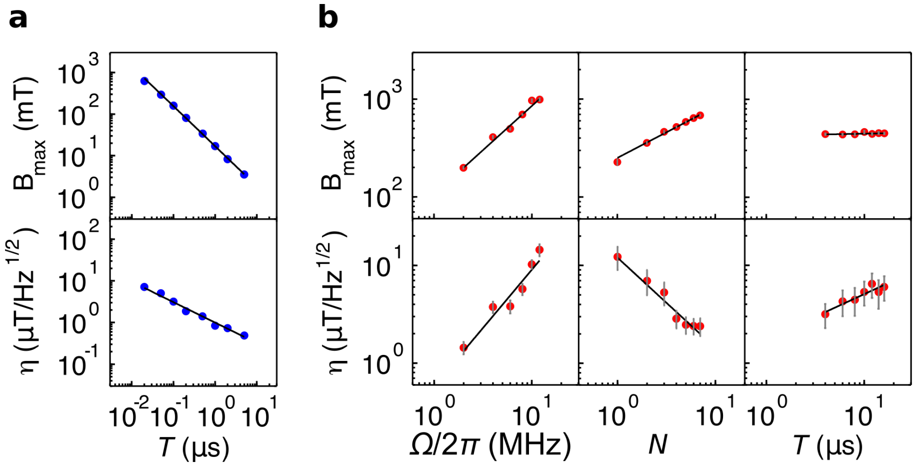

Supplementary Figure 8 | Scaling of sensitivity and field range with three control parameters. **a**, Measured sensitivity and field range of dynamic-phase magnetometry as a function of interaction time *T*. The theoretical model predicts $\eta \propto T^{-1/2}$ and $B_{max} \propto T^{-1}$, and the measurement gives $\eta \propto T^{-0.49(6)}$ and $B_{max} \propto T^{-0.96(2)}$. **b**, Measured sensitivity and field range of geometric-phase magnetometry as a function of Rabi frequency *Ω*, winding number *N*, and interaction time *T*. The theoretical model predicts $\eta \propto \Omega^1 N^{-1} T^{1/2}$ and $B_{max} \propto \Omega N^{1/2} T^0$, and the measurements give $\eta \propto \Omega^{1.2(5)} N^{-0.92(1)} T^{0.46(1)}$ and $B_{max} \propto \Omega^{0.9(1)} N^{0.52(1)} T^{0.02(1)}$. (**a**, **b**) Dots indicate data points and lines are linear fits to data.

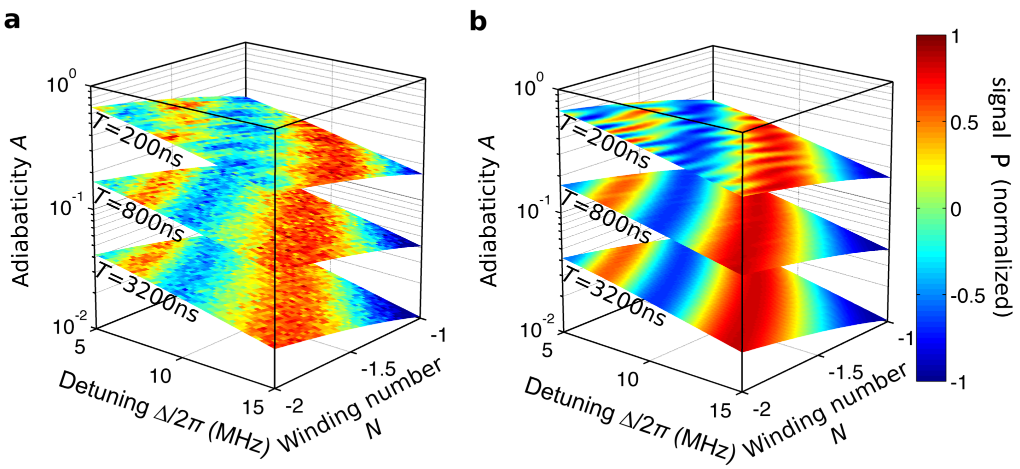

Supplementary Figure 9 | Measurement and simulation of geometric phase signal in the nonadiabatic regime. **a**, Measurement of the cosine of the geometric phase as a function of microwave frequency detuning Δ and winding number N with a fixed Rabi frequency of $\Omega/2\pi$ = 13 MHz. Three layers represent isochrone planes at T = 200, 800, 3200 ns. Vertical axis is adiabaticity $A = \dot{\rho}\sin\theta/2R$, where $R = (\Omega^2 + \Delta^2)^{1/2}$. The amplitude of each hyperfine oscillation is extracted by fitting the data along Δ at N = -2, T = 3200 ns to an analytical expression for the geometric-phase signal including the three NV hyperfine transitions. The measured signal is normalized to the mean of these three amplitudes. The analytical expression for the geometric phase signal assumes adiabaticity and thus is independent of the interaction time T. The data for layers at T = 3200 ns and 800 ns ($0.01 < A < 0.2$) look very similar, as expected. However, the layer at T = 200 ns ($A > 0.2$) looks distinctly different, indicating that the analytical expression becomes invalid in the nonadiabatic regime. **b**, Simulation of the cosine of the geometric phase including the three hyperfine transitions with the relative amplitudes obtained in **a** (see also Methods). The measurement and simulation agree, indicating that the time evolution of the spin state is described deterministically by the Schrödinger equation even in the nonadiabatic regime.
